# Effectiveness of Problem-Solving Therapy in Improving Patient Mental Health, Function, Quality of Life, and Mortality Post-Stroke: A Systematic Review

**DOI:** 10.3390/bs14060446

**Published:** 2024-05-25

**Authors:** Ha Thi Le, Kenta Honma, Hiroki Annaka, Shunxiang Sun, Tomonori Nomura

**Affiliations:** 1 Graduate School, Niigata University of Health and Welfare, Niigata 950-3198, Japan; hwd24021@nuhw.ac.jp; 2 Rehabilitation Department, Haiduong Medical Technical University, Haiduong 03117, Vietnam; 3 Department of Occupational Therapy, Faculty of Rehabilitation, Niigata University of Health and Welfare, Niigata 950-3198, Japan; kenta-honma@nuhw.ac.jp (K.H.); hiroki-annaka@nuhw.ac.jp (H.A.); nomura@nuhw.ac.jp (T.N.)

**Keywords:** problem-solving therapy, mental health, depression, anxiety, apathy, function, quality of life, mortality, stroke, systematic review

## Abstract

Problem-solving therapy (PST) is a potential psychological intervention aimed at preventing and treating psychological issues in stroke patients, although its efficacy is not clearly established. This systematic review assessed the effectiveness of PST in improving mental health, functioning, quality of life, and mortality in this population. Six databases were searched for literature indexed through March 2024, including the Cochrane Library, PubMed, Scopus, CINAHL, NeuroBITE, and OTseeker. This review (CRD42023483757) followed the PRISMA guidelines and the Cochrane Library Handbook, utilizing the RoB 2 tool and GRADE system to assess the quality of the evidence. It included eight randomized controlled trials (RCTs) involving 1249 patients with stroke. Among them, five RCTs showed that PST might improve depression. Additionally, individual RCTs demonstrated the efficacy of PST in addressing patient anxiety, apathy, and coping. With respect to mental health, PST might affect patient quality of life and mortality. However, the results of four RCTs demonstrated no effect of PST on patient functioning. The quality of evidence for the outcomes ranged from very low to high. PST may improve mental health, quality of life, and mortality in patients with stroke.

## 1. Introduction

Stroke is the second-leading cause of global mortality after ischemic heart disease and ranks among the top two contributors to disability worldwide, imposing significant burdens on individuals, families, societies, and nations [1,2]. Stroke can lead to changes in patients’ physical and psychological functioning. However, current treatments predominantly prioritize physical treatment, often overshadowing mental health considerations, resulting in a lack of attention and inadequate treatment of mental health issues [3,4,5,6]. People with stroke have a high rate of mental health issues, including anxiety, depression, psychosis, post-traumatic stress disorder (PTSD), apathy, personality changes, and difficulty coping emotionally [5,7,8,9,10,11]. These issues significantly affect their recovery, social reintegration, response to rehabilitation, and overall quality of life (QoL) [9,12]. Therefore, mental health is a challenge in patients who have experienced a stroke [13].

Addressing mental health problems, particularly depression and anxiety, requires a comprehensive approach that includes both pharmacological and non-pharmacological treatments [14]. Although pharmacological interventions are effective, they also have potential side effects, may be less effective when discontinued, and are not universally effective for all people with stroke [15,16,17]. Currently, insufficient evidence exists to support non-pharmacological treatments, particularly psychological interventions. In this context, problem-solving therapy (PST) has emerged as a potential therapeutic option.

PST, originally formulated by D’Zurilla and Goldfried [18,19], focuses on cultivating adaptive problem-solving attitudes and skills to alleviate and prevent psychopathology. The mechanism of PST is thought to be related to the activation of the amygdala, the center of emotion, motivation, and consciousness in the brain [20,21,22]. PST seeks to promote adaptive problem-solving by instilling an optimistic and confident attitude toward problems (positive problem orientation). It assists patients in internalizing four basic problem-solving skills, known as the PST protocol: first, defining the problem; second, brainstorming possible solutions; third, evaluating and selecting the most promising solution; and fourth, implementing the preferred solution and reflecting on the outcome [23]. Through these systematic steps, therapists enable individuals to effectively address problems, refine coping skills, and alleviate depressive symptoms [24,25,26,27,28]. Therefore, PST contributes to improved QoL and physical well-being [29]. Moreover, it can be delivered by health professionals without a psychological background, including nurses, therapists, community physicians, and social workers, following a short training period [30], thus making the therapy more accessible. PST can also be delivered as a tele-intervention, which has been shown to be acceptable, appropriate, and satisfactory for both patients and caregivers [31].

PST is an effective psychotherapeutic intervention for the treatment and rehabilitation of people with a range of conditions, including depression, anxiety, suicidality, personality disorders, brain injury, dementia, cancer, and diabetes mellitus [23,32,33,34,35,36]. Although evidence of its effectiveness in many conditions is robust, it is less conclusive in the context of stroke survivors. Clinical trials have investigated the effectiveness of PST, and systematic reviews and guidelines have mentioned PST or psychotherapy after stroke; however, they have reported conflicting findings without any clear evidence [6,37,38,39,40]. This raises the fundamental question of whether PST use in patients with stroke is justified and what outcomes can be expected. To the best of our knowledge, no systematic reviews have been conducted on the specific effects of PST in patients with stroke. Hence, this review aimed to systematically evaluate the effects of PST in stroke survivors to provide clearer evidence-based insights into the potential of PST in the prevention and treatment of mental health challenges following stroke.

## 2. Materials and Methods

This systematic review was performed following the Preferred Reporting Items for Systematic Reviews and Meta-analyses (PRISMA) guidelines [41,42] and the Cochrane Handbook for Systematic Reviews of Interventions [43]. The review protocol was registered in the International Prospective Register of Systematic Reviews (PROSPERO, registration number CRD42023483757) [44].

### 2.1. Search Strategy

We systematically searched six databases, including the Cochrane Library, PubMed, Scopus, CINAHL, NeuroBITE, and OTseeker, as well as the gray literature, for articles indexed before 26 October 2023, to identify English-language articles related to our topic. The search was updated on 14 March 2024. We also searched the reference lists of the included full-text articles.

The search strategy was based on the PICO tool recommended by the Cochrane Library [43]: (1) problem: stroke; (2) intervention: problem-solving therapy; (3) comparison: antidepressant therapy/placebo, usual care, or other therapies; and (4) outcome: any possible outcome, such as mental health, functioning, and QoL. Each database was searched for free-text and control vocabulary terms. The complete search strategy is described in Supplementary Material S1.

### 2.2. Study Selection and Inclusion Criteria

Two authors (T. N. and H. T. L.) independently performed the study selection process and assessed the eligibility of the articles based on the title, abstract, and full text according to the inclusion criteria. During the screening and selection processes, a third author (K. H.) was consulted to resolve any disagreements.

The inclusion criteria were (1) randomized controlled trials (RCTs); (2) studies in adults (aged >18 years) diagnosed with stroke; (3) studies using PST, either alone or in combination with other therapies; and (4) RCTs published in English. The exclusion criteria were RCTs with a study population that included patients with stroke and other medical conditions.

### 2.3. Data Extraction

Data from each of the included studies were independently extracted by two authors. Any disagreement was resolved by consulting a third author. Additionally, we attempted to contact key authors of the included studies via email for feedback and input as required, but regrettably, we did not receive any responses.

The following data were extracted from the included studies: (1) general study characteristics, including authors, publication year, study design, age, sex, type of stroke, sample size, number of groups, and inclusion criteria; (2) characteristics of the therapy in different groups, including the therapy type, session duration, days per week, and number of weeks; and (3) data related to outcomes, including the type of outcome, evaluations, follow-up time, and related quantitative data. Outcome measures were decided after including the studies and assessing the quality of the outcome scales.

We used Rayyan [45] to import data, remove duplicate studies, and screen titles and abstracts; Microsoft Excel for data extraction; and Endnote to manage the included studies and references.

### 2.4. Statistical Analysis

After data extraction and emailing, four and five studies, respectively, had outcomes related to depression and function; however, these studies reported results that were assessed at different follow-up times, and we did not have the original data. Although we emailed the authors to obtain this information, we did not receive a response; therefore, we could not perform a meta-analysis.

### 2.5. Assessment of the Quality of Evidence and the Risk of Bias

The risk of bias and the methodological quality were assessed independently by two reviewers (T.N. and H.T.L.). The risk of bias was evaluated using version 2 of the Cochrane Risk of Bias Tool for Randomized Trials (RoB 2). This scale comprises five items (randomization process, deviations from the intended interventions, missing outcome data, measurement of the outcome, and selection of the reported results) and classifies the risk of bias as “low risk” (+), “some concerns” (!), and “high risk” (−) [46]. Furthermore, the Grading of Recommendations Assessment, Development, and Evaluation (GRADE) approach was used to analyze the overall quality of evidence for each outcome. This system assesses various aspects for upgrading levels for domains such as large effect magnitude, confounding, and dose–response gradient, and for downgrading levels including the risk of bias of each study, inconsistency, indirectness, imprecision, and risk of publication bias [47,48].

### 2.6. Publication Bias

Due to the variability in outcomes among the eight RCTs included in this review and the differing numbers of RCTs for each outcome, it was not feasible to create funnel plots to assess for publication bias [43].

## 3. Results

### 3.1. Study Selection

**Figure 1:**
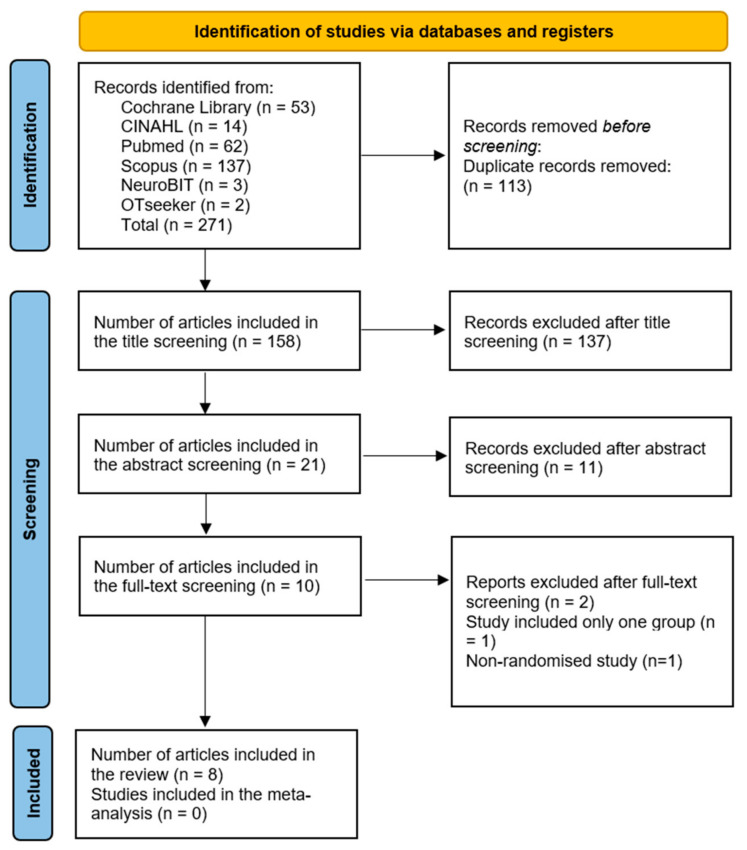
Preferred reporting items for systematic reviews and meta-analyses (PRISMA) 2020 flow diagram for the systematic review of the effect of problem-solving therapy in patients with stroke.

The flowchart based on the PRISMA guidelines (Figure 1) illustrates the results of the search process and study selection phases. Initially, 271 studies were identified from the six databases. Eight trials met the inclusion criteria and were included in the review after removing duplicates and screening the titles, abstracts, and full texts.

### 3.2. Main Characteristics of the Included Studies and Outcomes

A summary of the main characteristics and details of the PST interventions/sessions of the eight included trials is presented in Table 1. The eight studies included 1249 participants aged between 18 and 90 years. Of these, seven studies reported the sex of the participants, ranging from 31.8% to 61.7% male. The included trials consisted of five three-arm trials, four of which compared PST with escitalopram and a placebo group, while one trial compared nonspecific support with a volunteer support group. The remaining three trials compared two groups, with control groups including usual care, outpatient rehabilitation, and education groups. In eight studies, PST was used to intervene from the acute to chronic phase in stroke patients, and the follow-up period generally ranged between 6 weeks and 1 year (one study had a follow-up of 8 years). Seven RCTs mentioned exclusion criteria, including serious or unstable concurrent illness, patient inability to communicate, progressive neurodisorders, or severe comprehensive deficits, but most of the studies did not have any criteria about the severity of motor or cognitive function. No study mentioned caregiver involvement in interventions. Regarding the intervention context, three RCTs [49,50,51] mentioned that the intervention was at home.

### 3.3. Outcome Measures

After including the studies and assessing the quality of the outcome scales, the primary outcome evaluated was mental health. This was measured using various tools, including the Center for Epidemiological Studies Depression (CES-D) scale, the Diagnostic and Statistical Manual of Mental Disorders, fourth edition (DSM-IV), the 28-item General Health Questionnaire (GHQ-28), and the Present State Examination—Short Form (PSE) for depression. Additionally, anxiety was assessed using the generalized anxiety disorder (GAD) scale, apathy using the Apathy Scale, and coping strategies using the Coping Inventory for Stressful Situations (CISS). The other outcomes related to activities of daily living (ADL) and functions included the Functional Independence Measure (FIM), Barthel index, and Frenchay Activities Index (FAI). QoL was assessed according to the health-related quality of life (HRQoL), including Stroke-Specific Quality of Life (SS-QoL) and the EuroQol (EQ-5D–5L). Mortality based on death outcomes was also assessed.

Regarding treatment providers, five studies reported trained providers from different healthcare settings, including doctoral students, neuropsychologists, therapists, and psychiatrists.

Regarding the intervention, PST was delivered in 6–12 sessions, with four studies delivering 12 sessions using the same protocol (Table 1). The duration of each session was approximately 1–1.5 h and around one session per week, except for one study with one session every 2 weeks. One study used group therapy, which consisted of three to six participants in each group and included homework assignments.

All the studies reported no conflicts of interest.

### 3.4. Risk of Bias Assessment

**Figure 2:**
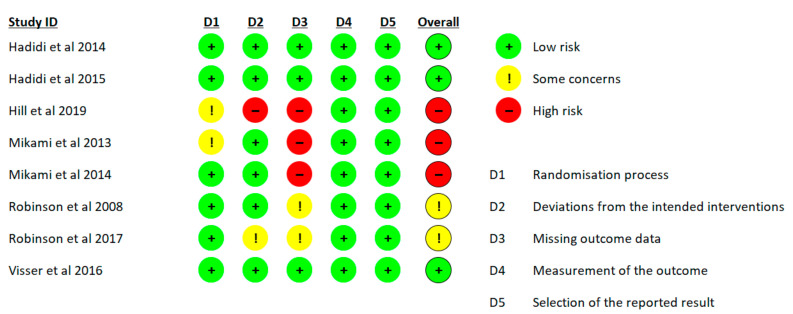
Risk of bias summary showing the determinations for each risk of bias item/domain (D) for each of the eight included studies [17,20,49,50,51,52,53,54].

The risk of bias ratings for all eight studies according to the Cochrane Handbook guidelines are shown in Figure 2.

**Figure 3:**
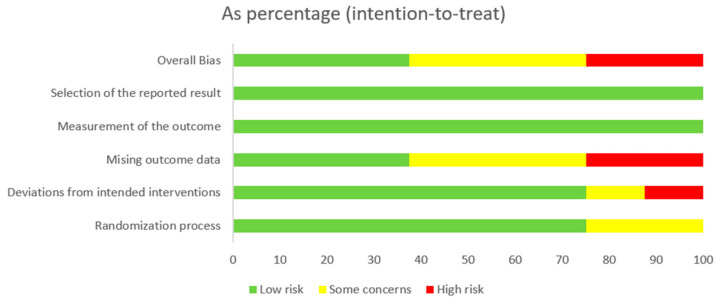
Risk of bias graph showing the determinations for each risk of bias domain for each of the eight included studies.

The distribution of the risk of bias across the studies is shown in Figure 3. Two studies had a high risk of bias, two studies were rated with some concerns, and three studies had a low risk of bias. These studies were graded into three domains: missing outcome data, deviations from the intended interventions, and randomization process. Regarding the measurement of the outcome and selection of the reported result criteria, all eight studies were assessed as having a low risk of bias. Both studies with high risks of bias and three studies with some concerns had missing data that could affect the results.

### 3.5. Certainty of the Evidence

The evidence quality of the problems according to the GRADE assessment is shown in Table 2, with only the coping outcome showing high-quality evidence. The outcomes of anxiety and QoL showed moderate-quality evidence due to missing outcome data (anxiety) and imprecision (QoL) due to the small sample size. With low-quality evidence, depression, apathy, and ADL were judged as risk factors for bias and inconsistency. Finally, the mortality outcome was judged to have very low-quality evidence because of the small sample size, leading to imprecision.

### 3.6. Effects of PST

The eight RCTs used different outcome measures. Regarding mental health, five studies showed that PST may affect depression [20,49,50,52,55]. Regarding other outcomes, one study each showed effectiveness for anxiety [54], apathy [53], and coping [50]. Among the remaining outcomes, four studies considered no effect on ADL; one study measured QoL, and one study assessed the relationship between PST and mortality, and both showed effectiveness. We discuss the evidence for these outcomes in greater detail.

#### 3.6.1. Effects of PST on Mental Health

Depression and anxiety were predominant mental disorders, while apathy and coping strategies were also mentioned.

Further, five studies [20,49,50,51,52] involving 616 patients reported the outcome of depression using different scales: three studies assessed CES-D (two studies used a short version of the CES-D with a cutoff value for probable depression of 5; one used a 20-item scale with a cutoff of 16), one study used the DSM-IV, while another study used the GHQ-28 and PSE scales. In general, over time periods ranging from three months to one year, four studies showed a possible effect of PST on depression, whereas one study showed no evidence of efficacy (Table 2).

One study using the GAD to diagnose anxiety disorder reported that the placebo group was 4.00 times (adjusted hazard ratio [HR]: 4.00; 95% confidence interval [CI]: 1.84–8.70; *p* = 0.0005) more likely to develop GAD compared to those who received PST after one year [54].

Apathy is a mental state characterized by a lack of motivation and a decrease in goal-directed behavior, goal-directed cognition, and goal-directed emotions [56,57]. One study assessed apathy using the Apathy Scale (clinician version) [58], reporting that PST demonstrated significantly greater effectiveness in preventing apathy onset compared to the placebo group during the initial year post-stroke [53].

Coping refers to the cognitive and behavioral strategies used to handle stressful situations and the associated emotions. Coping styles include (a) emotion-focused coping, (b) problem-focused or task-oriented coping, (c) active coping, (d) avoidant coping, (e) accommodative coping, and (f) assimilative coping [59,60]. One study assessed coping outcomes and reported a significant difference between the PST and control groups in terms of avoidant coping and task-oriented coping, with effect sizes of 0.33 and 0.43, respectively, 6 months after intervention. However, the improvements observed in the PST group were not maintained after 1 year [50].

#### 3.6.2. Effects of PST on Daily Life, QoL, and Mortality

ADL: Four studies (543 patients) reported the ADL of participants; three studies used the FIM to assess patients, while one study used the BI and FAI. All studies [49,51,52,53] reported no significant differences in function until one year after the intervention.

QoL: PST may improve general HRQoL recovery, exhibiting an effect size of 0.34 (*p* = 0.034) compared with the control group at 6 months post-intervention; however, this result was not significant at one year after intervention [50].

Mortality: Robinson et al. reported that compared with the placebo and escitalopram groups, the PST group exhibited a significant delay in mortality 8 years after stroke [17].

## 4. Discussion

This systematic review collected evidence regarding the effects of PST in patients with stroke. The results consisted of two main parts: first, evidence of a possible effect on mental health, including depression, anxiety, apathy, and coping, and second, there was evidence that there was no effect on ADL but that it had a positive effect on QoL and mortality. The quality of evidence for the outcomes varied from very low to high, according to the GRADE system. This is discussed in detail below.

### 4.1. Mental Health

Patients with stroke might experience many mental health challenges. This review collected the best evidence on the effects of PST in patients with stroke. The outcomes of the evidence focused on only four of these challenges: depression, anxiety, apathy, and coping. Four out of five studies showed that PST has a positive effect on depression; however, all five studies were rated as ‘low quality’ according to the GRADE system. Meanwhile, anxiety, apathy, and coping outcomes showed clear evidence that PST improved mental symptoms, with GRADE findings from low to high.

Evidence also suggests that psychological symptoms (depression, anxiety, apathy, and coping) affect functional disability, QoL, prognosis, and survival after stroke. Moreover, depression is closely associated with decreased physical and cognitive recovery. Anxiety and apathy often co-occur with depression, leading to delayed recovery from depression, delayed overall recovery, and increased likelihood of experiencing other diseases. Regarding coping, premorbid coping styles may be risk factors for PTSD, a common sequela among stroke survivors, and are also correlated with pain or discomfort at one year after stroke [8,12,59,61,62,63,64,65,66,67,68,69,70,71]. Thus, based on these relationships and the effect of PST on improving signs of depression, anxiety, apathy, and coping, PST might indirectly affect physical and cognitive recovery, function, QoL, and mortality, as some of these outcomes (QoL and mortality) were demonstrated in the present review.

Regarding post-stroke mental health, two studies [55,72] aligned with the present review regarding the reduction of symptoms regarding depression, anxiety, and QoL, but they were not included because they were not RCTs. PST also shows efficacy in significantly improving depression and anxiety in primary care patients with depression and/or anxiety [30] and depressive disorders in adults and young individuals [25,73].

Problem-solving training may help patients cope with personal difficulties; however, its effectiveness in reducing depressive symptoms may be limited [23]. In patients with depression, PST is as effective as medication treatments and other psychosocial therapies and is significantly more effective than support/attention control and no treatment [19]. Both pharmacological and psychotherapy are recommended as primary approaches for treating and preventing post-stroke depression [61,74]. The results of the present review showed that PST has unclear effects on depression and may need to be combined with other interventions for optimal and clear results.

Regarding the inclusion and exclusion criteria, three RCTs excluded patients with poor mental health (one RCT—depression [52]; one—anxiety [54] and one—apathy [53]). PST was used for prevention, and other RCTs used PST as an intervention to treat mental issues. No study has demonstrated the effectiveness of PST as a treatment after the diagnosis of mental health conditions in stroke. Therefore, future research should focus on the use of PST for the treatment of mental health conditions. Three studies assessed PST compared with drugs, each evaluating different outcomes, including depression [52], anxiety [54], and apathy [53]. Among them, escitalopram was more effective than PST, with a higher adjusted HR compared with placebo. However, when the medication was stopped for 6 months, new cases of major depression increased significantly within the escitalopram group, with no new diagnoses of depression in the PST or placebo groups [75]. Among the five RCTs reporting depression outcomes, the PST doses ranged from 6 to 12 sessions, with no differences in effects according to treatment duration. In a meta-analysis using PST for adult depression, extended PST with ten or more sessions showed a considerably greater effect size, although this difference was not statistically significant [73]. Only one study included an intervention follow-up group; however, the result was not significant (*p* = 0.577) [50]. As PST can be delivered by different healthcare workers without a psychological background, it has become easier to approach, feasible, effective, and safe to implement in clinical practice.

In summary, the results showed that PST had no clear effect but possibly an improvement in depression, with low-quality evidence. However, the studies demonstrated evidence of its effectiveness in anxiety, apathy, and coping. Therefore, PST should be considered as a potential psychological prevention and intervention for the promotion of general mental health in patients after stroke.

### 4.2. Function, QoL, and Mortality

Many factors affect function, especially ADL, including physical and psychological abilities. However, all four RCTs that used the FIM, BI, and FAI to assess function detected no significant differences [49,51,52,53]. Regarding mental health challenges, both apathy and depression were negatively associated with ADL [76]. Zhang et al. suggested that neuropsychiatric disorders after stroke negatively impact functional recovery [11]. In addition, the inclusion criteria in the included studies suggest the need for more specific definitions of mental problems at an early stage after stroke to allow for a deeper analysis and a more accurate assessment of the relationship between PST and functions related to mental health.

One RCT assessed the outcomes of QoL, and one assessed mortality; both showed positive results following the use of PST. Evidence has shown a relationship between psychological factors that lead to lower QoL after stroke and their effects on mortality [11,12,65,74,77,78,79,80,81,82]. The results of this review demonstrated that PST could improve mental health; therefore, it may indirectly improve QoL and mortality. In addition, patients with stroke report high satisfaction with PST [51,72]. Moreover, PST or improved social problem-solving ability has shown positive effects on QoL and reduced suicide risk or mortality in other conditions, such as dementia and mild cognitive impairment in primary care patients [83,84]. Although QoL was a secondary outcome in the included RCTs, with moderate quality of evidence, PST may be one of the factors associated with improved QoL after stroke. One study involving 122 patients evaluated 8 years after the intervention reported an increased time to death for those who received PST; however, the sample size was too small to assess the mortality outcome, making the GRADE assessment very low. Therefore, more evidence is needed.

In summary, PST showed no effect on ADL but had a positive effect on QoL and mortality.

### 4.3. Limitations and Suggestions

This review has a few limitations: (i) as the search was conducted in six databases and limited to English, not all available evidence may have been identified; (ii) while the authors were emailed to provide missing data, they did not respond; therefore, a meta-analysis could not be performed; and (iii) five of the eight studies had a risk of bias, which reduced the quality of the evidence. However, the findings of this systematic review have important implications for PST after stroke. First, in terms of research, (i) future research should be conducted more rigorously to obtain high-quality evidence, and (ii) PST in mental health treatment should be investigated in the field of stroke. Second, in terms of clinical application, due to its demonstrated effectiveness, lack of side effects, safety, and feasibility, PST appears to have a positive effect on mental health issues after stroke and may serve as a good non-pharmacological treatment that can be implemented early in the recovery process.

## 5. Conclusions

The results of this review suggest that PST may prevent mental health issues (including depression, anxiety, apathy, and coping) and improve mental health status and QoL, along with reducing mortality in patients with stroke. However, outcomes such as function and ADL did not show a clear benefit from PST itself. Based on the GRADE approach, the levels of evidence varied from very low to high. Therefore, these results should be interpreted with caution because of the potential risks of bias, imprecision, and missing data from the included RCTs. Additionally, more evidence is needed on the benefits of PST in mental health treatment in combination with pharmacological and other non-pharmacological treatments.

## Figures and Tables

**Table 1:** Main characteristics and outcomes of the included studies.

Author	Participant Demographics:Average Age (Range) and Sex	Groups (n)	Control Group	Interventions (Dose)	Follow-Up Time	PST Provider	Diagnosis Scale	Outcome Measures
Robinson et al. 2008 [52]	64.2 (50–90) years 35% male Stroke within 3 months	Escitalopram (n = 59) Placebo (n = 58) Non-blinded PST (n = 59)	− Escitalopram − Placebo (all pills were identical)	12 total sessions	2, 6, 12 months	Trained therapists	DSM-IV and HDRS	DSM-IV FIM
Mikami et al. 2013 [53]	63.9 (50–90) years 60.4% male Stroke within 3 months	Escitalopram (n = 51) Placebo (n = 47) Non-blinded PST (n = 56)	− Escitalopram − Placebo	12 total sessions	3, 6, 9, 12 months	-	Apathy Scale DSM-IV	AS FIM
Mikami et al. 2014 [54]	65 (50–90) years 61.7% male Stroke within 3 months	Escitalopram (n = 47) Placebo (n = 49) Non-blinded PST (n = 53)	− Escitalopram − Placebo	12 total sessions	3, 6, 9, 12 months	-	GAD DSM-IV (SCID)	GAD
Hadidi et al. 2014 [20]	No demographic differences between groupsIschemic stroke within 3 months to 2 years	PST (n = 5) Attention control (n = 5)	education weekly for 6 weeks (1 h/s)	6 sessions 1–1.5 h/s/w	6 weeks	Doctoral student	CES-D (cutoff 5–10 items)	CES-D
Hadidi et al. 2015 [49]	71 (55–89) years 31.8% male Ischemic stroke within the last 48 h	PST (n = 11) Control (n = 11)	Standard care/usual care	10 sessions 1.5 h/s/w.	5 weeks, 10 weeks, 3 months	Trained nursing doctoral student	CES-D (cutoff 5–10 items)	CES-D FIM
Visser et al. 2016 [50]	53.1 (18–75) years 53.01% male Stroke within 1 year (87.5% in the PST group, 79.5% in the control group)	PST (n = 88) Control (n = 78)	Outpatient rehabilitation alone	Group therapy (3–6 people). 8 s; 1.5 h/s/w, homework exercises.	10 days, 6 and 12 months	Trained neuropsychologist	CES-D (cutoff 16)	CISS CES-D SS-QoL EuroQoL EQ-5D-5L
Robinson 2017 [17]	64.18 (50–90) years 52% male Stroke	Escitalopram (n = 40) PST (n = 37) Placebo (n = 45)	− Escitalopram − Placebo	12 total sessions	8 years	-	DSM-IV (SCID) Deaths	Death
Hill et al. 2019 [51]	72 (65–79) years 54% male Stroke within the past month	PST (n = 151) Attention control (volunteers) (n = 149) Treatment-as-usual (n = 150)	− Treatment as usual − Volunteer: talking	6 s (6 h), 1 h/s/2w. ‘homework’	6, 12 months	Psychiatrist	GHQ-28	PSE-SF GHQ-28 BI, FAI
	18–90 years 31.8–61.7% male				6 weeks–1 year			

AS: Apathy Scale; BI: Barthel index; CES-D: Center for Epidemiological Studies Depression; CISS: Coping Inventory for Stressful Situations; Deaths (All): deaths due to any cause; EuroQol EQ-5D-5L; FAI: Frenchay Activities Index; FIM: Functional Independence Measure; GAD: generalized anxiety disorder; GHQ-28: 28-item General Health Questionnaire; HDRS: Hamilton-17 Depression Rating Scale; PSE-SF: Present State Examination—Short Form; SCID (DSM-IV): Structured Clinical Interview for DSM-IV (DSM-IV: Diagnostic and Statistical Manual of Mental Disorders); SS-QoL: Stroke-Specific Quality of Life Scale; h: hour; s: session; -: unknown; 12 total sessions: six treatment sessions over weeks 1, 2, 3, 4, 6, and 10, and six reinforcement sessions (months 4, 5, 6, 8, 10, and 12).

**Table 2:** Summary of findings: effect of problem-solving therapy compared with the control group for people with stroke.

Outcomes	Key Findings and Citations	Number of Participants (Studies)	Certainty of Evidence (GRADE)	Comments
**Mental health**				
Depression Scales: CES-D [20,49,50] DSM-IV [52] GHQ and PSE [51] Follow-up: 1 year	[20] 6 weeks: PST group: Median baseline 4 (0–10), decreased to 0 (0–9). Control group: median baseline 2 (1–4), increased to 3 (0–5). [50] 10 weeks: clinically significant difference for a new cutoff score of 5, decrease from 8.7 to 3.1. 3 months: no significant difference between groups. [51] 6 and 12 months: no significant difference between groups (*p* = 0.577). [53] 12 months: compared with the placebo group, a significantly lower rate of depression in the escitalopram (adjusted HR, 4.5; 95% CI, 2.4–8.2) and PST (adjusted HR, 2.2; 95% CI, 1.4–3.5) groups. For intention to treat, PST did not show significant results over placebo (adjusted HR, 1.1; 95% CI, 0.8–1.5). [52] 12 months: significantly lower GHQ-28 and median PSE scores.	616 (5 RCTs)	⨁⨁◯◯ Low ^a,c^	Possible effectiveness. More evidence is needed.
Anxiety [54] Scales: GAD Follow-up: 1 year	Placebo group 4.95 times more likely to develop GAD compared with the escitalopram group, and 4.00 times (adjusted HR: 4.00; 95% CI: 1.84–8.70) more likely compared with the PST group.	102 (1 RCT)	⨁⨁⨁◯ Moderate ^a^	Effectiveness
Apathy [53] Scale: AS Follow-up: 1 year	Placebo group was more likely to develop apathy 3.47 times (adjusted HR: 3.47, 95% CI: 1.79–6.73) than patients administered escitalopram and 1.84 times (adjusted HR: 1.84, 95% CI: 1.21–2.80) than patients administered PST.	103 (1 RCT)	⨁⨁◯◯ Low ^a,d^	Effectiveness
Coping [50] Scale: CISS Follow-up: 1 year	Primary outcome: task-oriented coping: 6 m; the PST group showed significant improvement (*p* = 0.008). ES = 0.43; 12 m: improvement remained (*p* = 0.060). Secondary outcome: avoidant coping differed significantly between groups (ES = 0.33) but was not maintained after 1 year (*p* = 0.581). Emotion-oriented coping showed no significant difference between groups.	166 (1 RCT)	⨁⨁⨁⨁ High	Effectiveness
**ADL, quality of life, and mortality**				
ADL Scales: FIM [49,52,53] BI and FAI [51] Follow-up: 1 year	No studies demonstrated significant differences in function between groups.	543 (4 RCTs)	⨁⨁◯◯ Low ^b^	Ineffectiveness
QoL [50] Scales: SS-QoL Follow-up: 1 year	6 months: significant differences between groups (*p* = 0.034), ES = 0.34. 12 months: equal HRQoL (*p* = 0.245).	166 (1 RCT)	⨁⨁⨁◯ Moderate ^d^	Possible effectiveness. More evidence is needed.
Mortality [17] Follow-up: 8 years	PST group: significantly delayed mortality post-stroke.	82 (1 RCT)	⨁◯◯◯ Very low ^a,c,e^	Effectiveness

AS: Apathy Scale; BI: Barthel index; CES-D: Center for Epidemiological Studies Depression; CISS: Coping Inventory for Stressful Situations; DSM-IV: Diagnostic and Statistical Manual of Mental Disorders; ES: effect size; EuroQol EQ-5D-5L; FAI: Frenchay Activities Index; FIM: Functional Independence Measure; GAD: generalized anxiety disorder; GHQ-28: 28-item General Health Questionnaire; HR: hazard ratio; HRQoL: health-related quality of life; SS-QoL: Stroke-Specific Quality of Life Scale; RCT: randomized clinical trial. ^a^ Downgraded one level due to risk of bias. ^b^ Downgraded two levels due to risk of bias. ^c^ Downgraded one level due to inconsistency. ^d^ Downgraded one level due to imprecision. ^e^ Downgraded two levels due to imprecision.

## Data Availability

Not applicable.

## Supplementary Materials

The following supporting information can be downloaded at https://www.mdpi.com/article/10.3390/bs14060446/s1; Supplementary Material S1.
