# Supplementary material for: Effectiveness of Problem-Solving Therapy in Improving Patient Mental Health, Function, Quality of Life, and Mortality Post-Stroke: A Systematic Review
Source: Behav Sci (Basel). 2024 May 25;14(6):446. doi: 10.3390/bs14060446 (PMC11201169; doi:10.3390/bs14060446)
Supplement: Supplementary file 1 [file behavsci-14-00446-s001.zip › behavsci-2985359-supplementary.pdf]

Searching strategy in 6 databases for effectiveness of Problem-solving therapy in stroke

1. CENTRAL search strategy (Cochrane Library)

#1 MeSH descriptor: [Cerebrovascular Disorders] explode all trees
#2 [mh Basal Ganglia Cerebrovascular Disease]
#3 [mh Brain Ischemia]
#4 [mh Intracranial Embolism and Thrombosis]
#5 [mh Intracranial Hemorrhages]
#6 [mh Stroke]
#7 [mh Hemiplegia]
#8 (stroke* or "post stroke" or poststroke or post-stroke or apoplex* or "cerebral vascular disease" or cerebrovasc* or CVA or SAH or hemipar* or hemipleg* or paresis or paretic) :ti,ab,kw
#9 #1 or #2 or #3 or #4 or #5 or #6 or #7 or #8 or #9
#10 PST or "Problem solving therapy" or "Problem-solving therapy"
#11 #9 and #10

2. PUBMED search strategy

#1 "Stroke"[Mesh] OR "Stroke, Lacunar"[Mesh] OR "Hemorrhagic Stroke"[Mesh] OR "Embolic Stroke"[Mesh] OR "Thrombotic Stroke"[Mesh] OR "Ischemic Stroke"[Mesh] OR "Stroke Rehabilitation"[Mesh] OR "Infarction, Posterior Cerebral Artery"[Mesh] OR "Brain Stem Infarctions"[Mesh] OR "Infarction, Middle Cerebral Artery"[Mesh] OR "Infarction, Anterior Cerebral Artery"[Mesh]

#2 (stroke* or "post stroke" or poststroke or post-stroke or apoplex* or "cerebral vasc*" or cerebrovasc* or cva or SAH or hemipar* or hemipleg* or paresis or paretic).ti,ab.
#3 #1 or #2
#4 (PST or "Problem solving therapy" or "Problem-solving therapy").ti,ab.

#5 #3 and #4

3. SCOPUS search strategy

TITLE-ABS-KEY ((stroke OR {post stroke} OR poststroke OR post-stroke OR apoplex! OR "cerebral vasc!" OR cerebrovasc! OR cva OR sah OR hemipar! OR hemipleg! OR paresis OR paretic OR {Ischemic stroke} OR {cerebral infarction} OR {cerebral thrombosis} OR {lacunar stroke} OR {Cerebral hemorrhage} OR {intracerebral hemorrhage} OR ich OR {subarachnoid hemorrhage} OR {brain hemorrhage}) AND (pst OR {Problem solving therapy} OR {Problem-solving therapy}))

4. CINAHL search strategy

TI Title
(((MH "Cerebrovascular Disorders+") OR (MH "Basal Ganglia Cerebrovascular Disease+") OR (MH "Cerebral Ischemia+") OR (MH "Carotid Artery Diseases+") OR (MH "Intracranial Arterial Diseases+") OR (MH "Arteriovenous Malformations+") OR (MH "Intracranial Embolism and Thrombosis+") OR (MH "Intracranial Hemorrhage+") OR (MH "Stroke+") OR (MH "Vertebral Artery Dissections+") OR (MH "Hemiplegia+"))

OR
(stroke* or “post stroke” or poststroke or post-stroke or apoplex* or “cerebral vasc*” or cerebrovasc* or cva or SAH or hemipar* or hemipleg* or paresis or paretic))
AND
(PST or “Problem solving therapy” or “Problem-solving therapy”)

Advanced search:

Boolean/phrase

English Language

Age Groups: all adult

Language: English

5. NeuroBITE search strategy
NeuroBITE (previously PsycBITE)
Language: English
Keyword: Problem solving therapy
Neurological Group: Stroke / CVA (Cerebrovascular Accidents)
Age group: Adults

6. OTseeker search strategy
Title/Abstract
(stroke or poststroke or CVA or cerebral vascular accident OR Ischemic stroke or cerebral infarction or cerebral thrombosis or cerebral embolism or lacunar stroke OR Cerebral hemorrhage or intracerebral hemorrhage or ICH or subarachnoid hemorrhage or SAH or brain hemorrhage)
AND

(“PST” or “Problem solving therapy” or “Problem-solving therapy”)
